# ADAM10 Negatively Regulates Neuronal Differentiation during Spinal Cord Development

**DOI:** 10.1371/journal.pone.0084617

**Published:** 2014-01-03

**Authors:** Xin Yan, Juntang Lin, Venkata Ajay Narendra Talabattula, Carolin Mußmann, Fan Yang, Andreas Wree, Arndt Rolfs, Jiankai Luo

**Affiliations:** 1 Albrecht-Kossel-Institute for Neuroregeneration, School of Medicine University of Rostock, Rostock, Germany; 2 Key Laboratory for Medical Tissue Regeneration of Henan Province, Xinxiang Medical University, Xinxiang, P.R. China; 3 Institute of Anatomy I, School of Medicine University of Jena, Jena, Germany; 4 Institute of Anatomy, School of Medicine University of Rostock, Rostock, Germany; Universitat Pompeu Fabra, Spain

## Abstract

Members of the ADAM (a disintegrin and metalloprotease) family are involved in embryogenesis and tissue formation via their proteolytic function, cell-cell and cell-matrix interactions. ADAM10 is expressed temporally and spatially in the developing chicken spinal cord, but its function remains elusive. In the present study, we address this question by electroporating ADAM10 specific morpholino antisense oligonucleotides (ADAM10-mo) or dominant-negative ADAM10 (dn-ADAM10) plasmid into the developing chicken spinal cord as well as by in vitro cell culture investigation. Our results show that downregulation of ADAM10 drives precocious differentiation of neural progenitor cells and radial glial cells, resulting in an increase of neurons in the developing spinal cord, even in the prospective ventricular zone. Remarkably, overexpression of the dn-ADAM10 plasmid mutated in the metalloprotease domain (dn-ADAM10-me) mimics the phenotype as found by the ADAM10-mo transfection. Furthermore, in vitro experiments on cultured cells demonstrate that downregulation of ADAM10 decreases the amount of the cleaved intracellular part of Notch1 receptor and its target, and increases the number of βIII-tubulin-positive cells during neural progenitor cell differentiation. Taken together, our data suggest that ADAM10 negatively regulates neuronal differentiation, possibly via its proteolytic effect on the Notch signaling during development of the spinal cord.

## Introduction

The spinal cord of vertebrates develops from the posterior neural tube, which differentiates along dorso-ventral and rostro-caudal axes and forms a coordinated structure [1], [2], where the specification and differentiation of distinct cell types are precisely controlled by a variety of morphogens, such as Sonic hedgehog (Shh), bone morphogenic protein (BMP) and Wnt molecules [3]–[5]. Shh secreted from the notochord and the floor plate forms a concentration gradient from ventral to dorsal in the developing spinal cord, while BMP and Wnts secreted from the roof plate create a concentration gradient from dorsal to ventral [6]. In addition to these morphogens, Notch signaling is essential for cell fate determination and controls processes of neurogenesis [7], [8] through its targeting hairy/enhancer of split (Hes) genes [9], [10]. In the developing mouse spinal cord, Notch1 and Notch3 are mainly expressed in the ventricular zone, and Notch2 in the floor plate [11]. Interestingly, Notch1 signaling is selectively responsible for the differentiation of interneurons in the V_2_ domain and of motoneurons in the V_MN_ domain [7], [11].

ADAM10, a member of the ADAM (a disintegrin and metalloprotease) family, is widely expressed in the brain, the spinal cord, and the visual system during development [12]–[16]. It is involved in protein proteolysis as well as cell-cell and cell-matrix interactions, thereby controlling neurogenesis and tissue formation [17], [18]. Based on its enzymatic activity, ADAM10 can shed cadherins (Cad) including E-Cad and N-Cad [19], [20]. ADAM10 regulates excitatory synapses through N-Cad cleavage [21] and is responsible for S2 cleavage of the Notch receptor, which is essential for neural progenitor cell maintenance [22]. Conditional deletion of ADAM10 in mice induces a precocious neuronal maturation, resulting in disruption of the neocortex and ganglionic eminence [23].

Previous studies have shown that ADAM10 is expressed in the developing spinal cord at both mRNA and protein levels [12], [14]. However, its precise functional role in spinal cord development is still unknown. In the present study we have investigated how ADAM10 regulates neuronal differentiation and other genes expression in vivo by electroporating ADAM10 morpholino antisense oligonucleotides (ADAM10-mo) or a dominant-negative ADAM10 mutant in the metalloprotease domain plasmid (dn-ADAM10-me) into the chicken spinal cord, as well as by in vitro cell culture investigation. Our data suggest that downregulation of ADAM10 drives differentiation of neural progenitor cells and radial glial cells into neurons, possibly via a proteolytic effect on the Notch signaling in the developing spinal cord.

## Materials and Methods

### Chicken Embryos

Fertilized eggs of White Leghorn chicken (*Gallus gallus*) were purchased from a local farm and incubated in a forced-draft incubator (Ehret, Emmendingen, Germany) at 37°C with 65% humidity. The stage of chicken embryo (HH) was evaluated according to the method of Hamburger and Hamilton [24]. For immunohistochemistry and in situ hybridization, the electroporated embryos were washed with Hepes-buffered salt solution (HBSS) and fixed with 4% formaldehyde solution (in HBSS) in ice for 8 hours or over night, according to the size of the embryos. Then samples were immersed in a serial of sucrose solutions (12%, 18% and 25% in HBSS). Finally, the samples were embedded in a Tissue-Tec O.C.T. compound (Science Services, Munich, Germany), frozen in liquid nitrogen and stored at −80°C for cutting.

### ADAM10 Morpholino Antisense Oligonucleotides and Plasmids

The ADAM10 morpholinos (ADAM10-mo) with the targeting sequence of 5′-ggattatcgtcctcgctagatccat-3′ and the standard negative-control morpholinos (Fluo-mo) of 5′-cctcttacctcagttacaatttata-3′ were purchased from the Gene Tools (Philomath, USA). This ADAM10-mo can specifically repress ADAM10 protein translation in chicken embryos [12], [25]. Both morpholinos were labeled with fluorescein at the 3′-end of the oligonucleotides in order to trace transfected cells after in vivo electroporation.

Chicken pCAGGS-ADAM10 containing the full-length sequence of chicken ADAM10 described previous [13], [14] was used as positive control for Western blot analysis.

The dn-ADAM10-me plasmid mutated in the metalloprotease domain by altering one amino acid was kind gift from Dr. C.A. Erickson (University of California, CA, USA) [12]. This dn-ADAM10-me contains a green fluorescent protein (GFP) as a reporter gene and the pCAGGS-GFP was used as a control.

To down-regulate human ADAM10 in cultured cells, the ADAM10 siRNA target sequence of 5′-gcgattgatacaatttacc-3′ was chosen according to the siRNA Target Finder (https://www.genscript.com/ssl-bin/app/rnai) and the synthetic oligonucleotides containing the siRNA target sequence from a commercial company (MWG, Berlin, Germany) were inserted into a hairpin siRNA expressing vector pGSHIN2-GFP driven by a human H1-RNA promoter (kind gift from Dr. Shin-ichiro Kojima, Feinberg School of Medicine Northwestern University, USA) for construction of the pGSHIN2-ADAM10 plasmid. pGSHIN2-GFP plasmid was used as a control.

### In vivo Electroporation in Chicken Embryos

Electroporation was carried out in the spinal cord of embryos at the incubation day 2 (E2; HH13) or E4 (HH24) according to the protocol described previously [26], [27]. In brief, after the morpholinos with a final concentration of 500 µM in phosphate buffer solution (PBS) or the dn-ADAM10-me plasmid (2 µg/µl) in the HBSS was injected into the neural tube, electroporation was performed by placing the electrodes on both sides of the embryos for in ovo electroporation (electrode CUY 610-P4, Nepa Gene, Chiba, Japan) at E2 or for ex ovo electroporation (electrode CUY 661–3_7, Nepa Gene) at E4 with the parameters (14 V, 60 ms pulse length, six pulses, 100 ms intervals) by an electroporator (CUY21-Edit; Nepa Gene). The electroporated embryos were further incubated for two days and collected for investigation at E4 or E6.

### Immunohistochemistry Analysis

Fluorescent immunostaining was performed on cryosections of the spinal cord according to the protocol described previously [28]. Primary rabbit polyclonal antibody against ADAM10 (AB19026, Chemicon), primary mouse monoclonal antibodies raised against nestin (ab92391, Abcam), viementin (3CB2), even-skipped homeobox 1 (Evx1; 99.1-3A2), ISL LIM homeobox 1 (Islet1; 40.3A4), LIM homeobox protein 3 (Lim3; 67.4E12), Mnx2 homeodomain protein (MNR2; 81.5C10), Neuron Specific Nuclear Protein (NeuN; MAB377, Chemicon), NK6 transcription factor-related locus 1 (NKx6.1; F55A10), NK2 transcription factor-related locus 2 (NKx2.2; 74.5A5), paired box gene 6 (Pax6), and Pax7 were used. Antibodies of 3CB2, 99.1-3A2, 40.3A4, 67.4E12, 81.5C10, F55A10, 74.5A5, Pax6, and Pax7 were kindly provided by the Developmental Studies Hybridoma Bank (DSHB) at the University of Iowa, USA. Appropriate Cy3-labeled secondary antibodies (Dianova, Hamburg, Germany) were used. Fluorescence was imaged under a fluorescence microscope (BX40; Olympus, Hamburg, Germany) equipped with a digital camera (DP70; Olympus) or a fluorescent microscopy system (BZ-8000; Keyence Deutschland GmbH, Neusenburg, Germany).

### In situ Hybridization

In situ hybridization on cryosections was performed as described previously [29]. Digoxigenin-labeled sense and antisense cRNA probes were transcribed using purified DNA plasmids as template according to the manufacturer’s instructions (Roche, Mannheim, Germany). The Hes1 and Hes5 plasmids were kind gifts from Dr. N. Jing (Shanghai Institutes for Biological Sciences, Shanghai, China) [30] and Dr. H. Nakamura (Tohoku University, Sendai, Japan) [31], respectively. The plasmids of glioma-associated oncogene homolog (Gli; Gli1, Gli2 and Gli3) were kindly obtained from Dr. Tabin (Department of Genetics, Harvard Medical School, Boston, USA) [32], [33].

### Apoptosis Analysis

Terminal deoxynucleotidyl transferase dUTP nick end labeling (TUNEL) assay was carried out to investigate cell apoptosis on cryostat sections using the In Situ Cell Death Detection Kit/TMR red (Roche) according to the manufacturer’s instructions.

### Cell Proliferation Assay with Bromodeoxyuridine Labeling

For the cell proliferation assay, 3 µl of 50 mM bromodeoxyuridine (BrdU; Sigma, Munich, Germany) in PBS was injected into chicken embryos through chorioallantoic vessels 2 hours before fixation. For BrdU detection, cryostat sections of 20 µm thickness were incubated in 2N HCl for 30 min followed by 0.1 M TBS buffer (pH 7.4). After pre-incubation with a blocking solution (5% skimmed milk and 0.3% Triton X-100 in tris-buffer solution) at room temperature for 60 min, the sections were incubated overnight at 4°C with the primary mouse monoclonal antibodies raised against BrdU (G3G4; from DSHB), followed by the secondary antibody at room temperature for 60 min.

### Cell Quantitative Analysis in Electroporated Immunostaining Sections

To compare neural proliferation and differentiation between the electroporated and unelectroporated side in the spinal cord, we counted the number of domain specific marker positive for neural progenitor cells and neurons overlapping with nuclear DAPI staining in the magnificent fluorescent immunostaining image within about 0.145 mm^2^ area under a Keyence BZ 8000 microscope. For counting cells, 2 images were taken from one section, 3 cryosections from each embryo were analyzed. At least 3 independent embryos for each group were evaluated.

### Cell Culture and Plasmid Transfections

The human neural progenitor cell line (ReNcell VM cell; ReNeuron Ltd, Guildford, UK) is derived from the ventral midbrain of a 10-week human fetus and immortalized by v-myc transduction. ReNcell VM cells were cultivated essentially as described previously [34]. Briefly, cells were cultured on laminin (Trevigen, Gaithersburg, MD, USA) coated flasks or 6-well plates in cultivation media, consisting of DMEM/F12 supplemented with B27 media supplement, GlutaMax Solution, gentamycin and heparin sodium salt (all materials come from Invitrogen, Karlsruhe, Germany). For cell proliferation, epidermal growth factor 2 (EGF2; Roche, Mannheim, Germany) with a final concentration of 20 ng/ml and basic fibroblast growth factor (bFGF; Roche) of 10 ng/ml was added in the medium. Differentiation was induced by washing the cells with HBSS, and followed by adding the medium without growth factors FGF2 and EGF.

Transfection of ReNcell VM cells was performed as the protocol described previously [35]. In brief, pGSHIN2-ADAM10 plasmid (4 µg/µl) for ADAM10 downregulation was transfected into the proliferating cells using the Amaxa Nucleofector System (Lonza Cologne GmbH, Germany) according to the manufacturer’s instructions. pGSHIN2-GFP was used as a negative control.

### Western Blot Analysis

Two days after electroporation, the fresh spinal cord was isolated from chicken embryos and separated into transfected and untransfected side under the fluorescent microscope according to the protocol described previously [36] (see Fig. S2). Then the separated transfected or untransfected spinal cords from 5 to 7 independent embryos were mixed together to form one sample for Western blot analysis. At least, three samples of the separated transfected or untransfected spinal cords from totally 20 embryos were collected for Western blot analysis. Protein concentrations were determined by a photometer (Tecan, Männedorf, Germany) using a Pierce bicinchoninic acid (BCA) protein assay kit (Therma Scientific). Equal amounts of protein (60 µg) from the samples were loaded on 4–15% sodium dodecyl sulfate (SDS)-polyacrylamide gels (Bio-Rad) and electrophoresis (PAGE) was performed. The protein bands were then electro-transferred from gel onto nitrocellulose membrane (GE Healthcare). The protein bands were then electro-transferred from gel onto nitrocellulose membrane (GE Healthcare). After the membrane was blocked with 3% milk in TBST Buffer for 1 h, the primary antibody was incubated with membrane at 4°C overnight, and then Alexa Fluor 680-coupled goat antirabbit IgG antibody (Molecular Probes) for ADAM10 detection, IRDye 680-coupled goat anti-rabbit IgG (LI-COR Biosciences) for cleaved Notch1, and the IRDye800-coupled goat anti-mouse IgG antibody (Rockland) for glyceraldehyde 3-phosphate dehydrogenase (GAPDH) detection were added on the blotting membrane for 2 h at room temperature. Detection was carried out using the Odyssey Infrared Imaging System (LI-COR Biosciences GmbH).

The Western blot analysis in ReNcell VM cells was performed according to the protocol described previously [37]. The following primary antibodies were used: rabbit anti-ADAM10 (AB19026; Millipore); rabbit anti-cleaved Notch1 (#2421; Cell Signaling); rabbit anti-Hes5 (sc-25395; Santa Cruz); and mouse anti-GAPDH (6C5; Abcam). The following secondary antibodies were used: Alexa Fluor 680-labeled goat anti-rabbit and IRDye 800-labeled goat anti-mouse antibodies (both obtained from Invitrogen). Visualization and quantification were performed with Odyssey Infrared Imaging System (LI-COR Biosciences GmbH, Bad Homburg, Germany). GAPDH was used as a loading control.

### Statistical Analysis

All data were presented as mean ± SEM. Statistical evaluation was carried out using the two-tailed Student’s t-test with Excel software (Microsoft, USA). A difference was considered to be significant when *p*-value is less than 0.05 (*p<0.05; **p<0.01; ***p<0.001).

## Results

**Figure 1:**
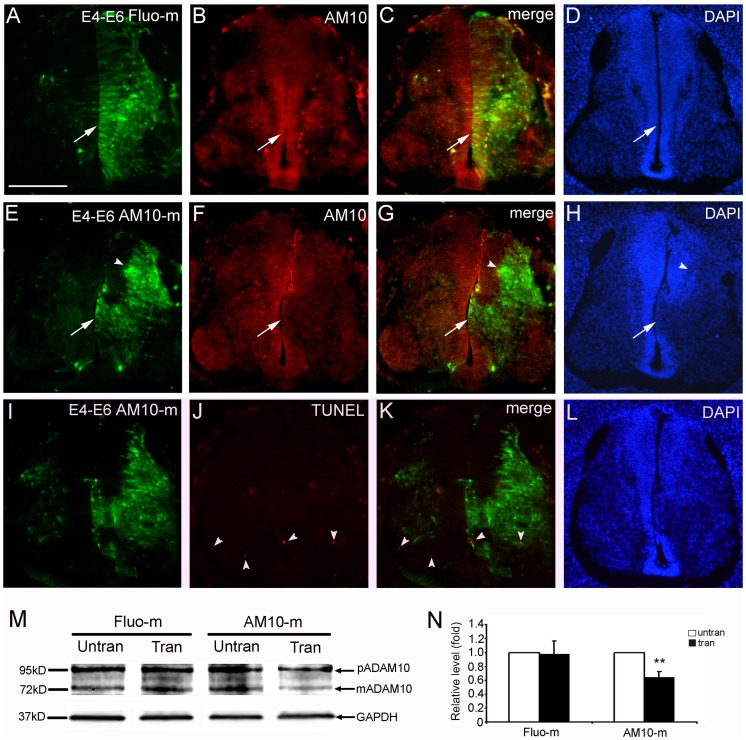
Electroporated ADAM10 morpholinos induce donwregulation of endogenous ADAM10 protein during development of the spinal cord. After electroporation at E4, the embryos were collected and transverse sections were cut at E6. The transfected cells are marked by green fluorescence. The control morpholinos and the untransfected side of the spinal cord serve as controls. The immune reactive cells are stained red. Cell nuclei are stained with 4′,6-diamidino-2-phenylindole (DAPI) as blue. (A–H) The expression of ADAM10 protein in the spinal cord transfected with control morpholinos (Fluo-m; A–D) or ADAM10 morpholinos (AM10-m; E–H). Arrows indicate transfected regions in the ventricular zone, and arrowheads in the mental layer. (I–L) Apoptotic cells (red, arrowheads) measured by TUNEL assay. (M, N) Representative Western blots (M) and semi-quantitative Western blot analysis (N) of ADAM10 protein, including a pre-mature (pADAM10) and a mature (mADAM10) form, in the transfected (tran) and untransfected side (untran) of ADAM10-morpholinos (AM10-m) or control morpholinos (Fluo-m) transfected embryos. GAPDH is used as a loading control. The amount of ADAM10 protein is normalized by the number of the control side, which is set to be 1. All data are presented as mean ± SEM from at least 3 independent samples (**p<0.01 compared to control). Scale bar, 200 µm in (A) for (A–L).

**Figure 2:**
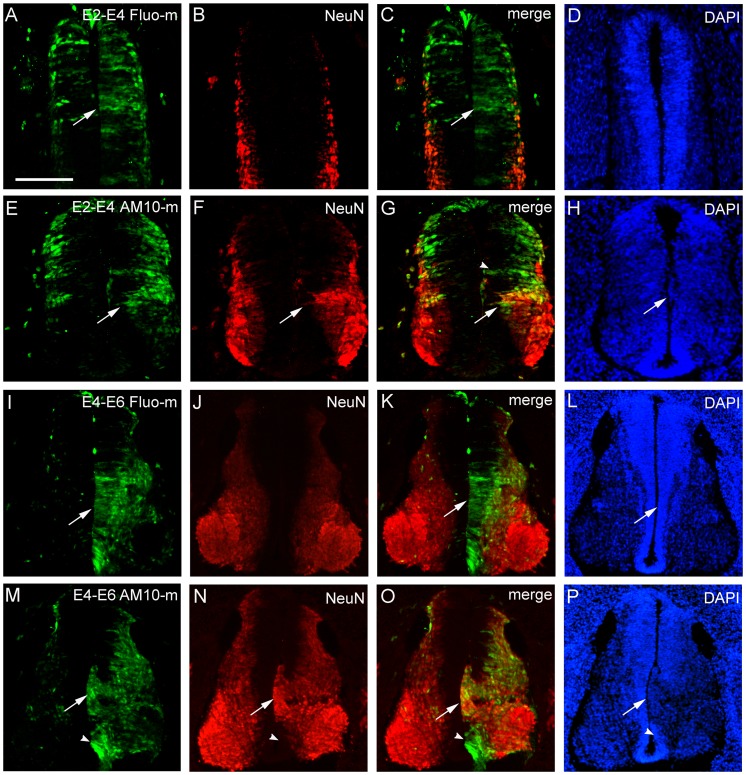
Downregulation of ADAM10 expression promotes neuronal differentiation in the developing spinal cord. Two days after electroporation, the embryos were collected and transverse sections cut at E4 or E6. The transfected cells are marked by green fluorescence. The control morpholinos and the untransfected side of the spinal cord serve as controls. The immune reactive cells are stained red. Cell nuclei are labeled blue with 4′,6-diamidino-2-phenylindole (DAPI). (A–P) NeuN immunostaining (red) in sections transfected with control morpholinos (Fluo-m; A–D, I–L) and ADAM10 morpholinos (AM10-m; E–H, M–P) at E2 (A–H) or E4 (I–P), respectively. Arrows indicate the transfected region in the ventricular zone of the basal plate, arrowheads in (G) of the alar plate and arrowheads in (M–O) above the floor plate. Scale bar, 200 µm in (A) for (A–P).

During development of the chicken spinal cord, the endogenous ADAM10 is spatiotemporally expressed. At E3, ADAM10 expression is strong in the ventricular zone and the floor plate at both mRNA and protein level [12], [14]. At E4, ADAM10 signals are maintained strongly in the ventricular zone and the lateral column of motoneurons [12], [14]. At E6, ADAM10 is predominant in the ventricular zone, especially the ventral part, and in the lateral motor column (Fig. 1B, C) [12], [14]. In the present study, we disturbed endogenous ADAM10 protein by transfecting the fluorescein labeled ADAM10-mo or the dn-ADAM10-me plasmid into the chicken spinal cord in the lumbar level at E2 or E4 by in vivo electroporation, and investigated the effect of ADAM10 downregulation on neuronal differentiation two days later at E4 or E6 by immunohistochemistry and in situ hybridization. The fluorescein labeled in the 3′-end of ADAM10-mo and the GFP protein were used as reporter markers to trace the ADAM10-mo and dn-ADAM10-me transfected cells, respectively. Both ADAM10-mo and dn-ADAM10-me have been identified to efficiently and specifically repress ADAM10 protein translation in chicken embryos [12], [25]. The construct of dn-ADAM10-me, in which the metalloprotease active-site glutamic acid (E385) is changed to an alanine (A), can efficiently block the ADAM10 metalloprotease function [12], [38]. The morpholinos were easily transfected into both the lumbar sides at E2 by in vivo electroporation (e.g., green in Fig. 2A, E), but predominantly into one side at E4 (e.g., green in Fig. 1A, E, I). Therefore, we mainly focused on E4-transfected embryos and compared the ADAM-mo transfected side (hereafter in the right side of the sections) to the untransfected side (left side), which showed the endogenous expression pattern.

### Downregulation of ADAM10 Induces Precocious Neuronal Maturation

First, we identified whether ADAM10-mo can efficiently downregulate the expression of endogenous ADAM10 in the developing spinal cord. When the control Fluo-mo was transfected alone at E4, the expression of the ADAM10 protein (red, Fig. 1B, C) between the transfected side (green, Fig. 1A, C) and the untransfected side (left side in Fig. 1A–D) at E6 was similar, although the fluorescein-positive (transfected) cells were observed in both the ventricular zone (arrows in Fig. 1A–D) and the mantle layer. In contrast, when ADAM10-mo was transfected, the expression of ADAM10 protein (red, Fig. 1F, G) in the transfected region (green, Fig. 1E, G) was decreased (arrows in Fig. 1F, G), when compared to the untransfected side (left side in Fig. 1E–H), suggesting that ADAM10-mo can efficiently downregulate ADAM10 protein expression in vivo, consistent with the previous studies [12], [25]. Interestingly, the prospective ventricular zone (arrow in Fig. 1H) disappeared when transfected with ADAM10-mo (arrows in Fig. 1E–H). Furthermore, Western blot analysis also showed that the amount of ADAM10 protein, including a pre-mature (pADAM10, Fig, 1M) and a mature form (mADAM10, Fig. 1M), was decreased in the ADAM10-mo transfected side (AM10-m, tran in Fig. 1M, N), when compared to the untransfected side (AM10-m, untran in Fig. 1M, N), the control group tissues with (Fluo-m, tran in Fig. 1M) or without (Fluo-m, untran in Fig. 1M) transfection. The separated pCAGGS-ADAM10 transfected spinal cords were used as a positive control for ADAM10 protein determination in Western blot analysis (data not shown). The quantitative results revealed that the difference of ADAM10 protein between the transfected side and the untransfected side in the ADAM10-mo transfected group was significant (*p* = 0.0035), but not in the control Fluo-mo group (*p* = 0.93).

To evaluate whether ADAM10-mo induces cell apoptosis in the spinal cord after electroporation, terminal deoxynucleotidyl transferase dUTP nick end labeling (TUNEL) assay was performed in the ADAM10-mo transfected sections at E6. The results showed that the number of apoptotic cells (arrowheads in Fig. 1J, K) on the transfected and untransfected sides was similar, suggesting that ADAM10-mo does not induce cell apoptosis in the developing spinal cord.

As next step, we investigated the effect of ADAM10 on neuronal differentiation using an antibody NeuN, a marker for postmitotic neurons. When the control Fluo-mo was transfected along at E2 (Fig. 2A–D) or E4 (Fig. 2I–L), the NeuN expression (red in Fig. 2B, C, J, K) in the mantle layers of both the lumbar sides was similar. In contrast, in ADAM10-mo transfected sections, NeuN protein (red, Fig. 2F, G, N, O) was strongly and specifically induced in the transfected regions of the base plate, even in the prospective ventricular zone (arrows in Fig. 2E–H, M–P), where a lot of fluorescein-positive cells were located (green, arrows in Fig. 2E–H, M–P) and the ventricular zone was defective at E4 (arrow in Fig. 2H) or absent at E6 (arrow in Fig. 2P). These data suggested that downregulation of ADAM10 induces precocious neuronal maturation, even in the prospective ventricular zone, where neural progenitor cells normally locate. However, transfected cells in the alar plate (e.g., green, arrowhead in Fig. 2G) or above the floor-plate (e.g., green, arrowhead in Fig. 2O) did not coexpress NeuN protein, suggesting that ADAM10 was involved in the neural differentiation in the specific region of the lumbar cord (see below for details).

### Downregulation of ADAM10 Alters Specifically Ventral Domain Patterns

**Figure 3:**
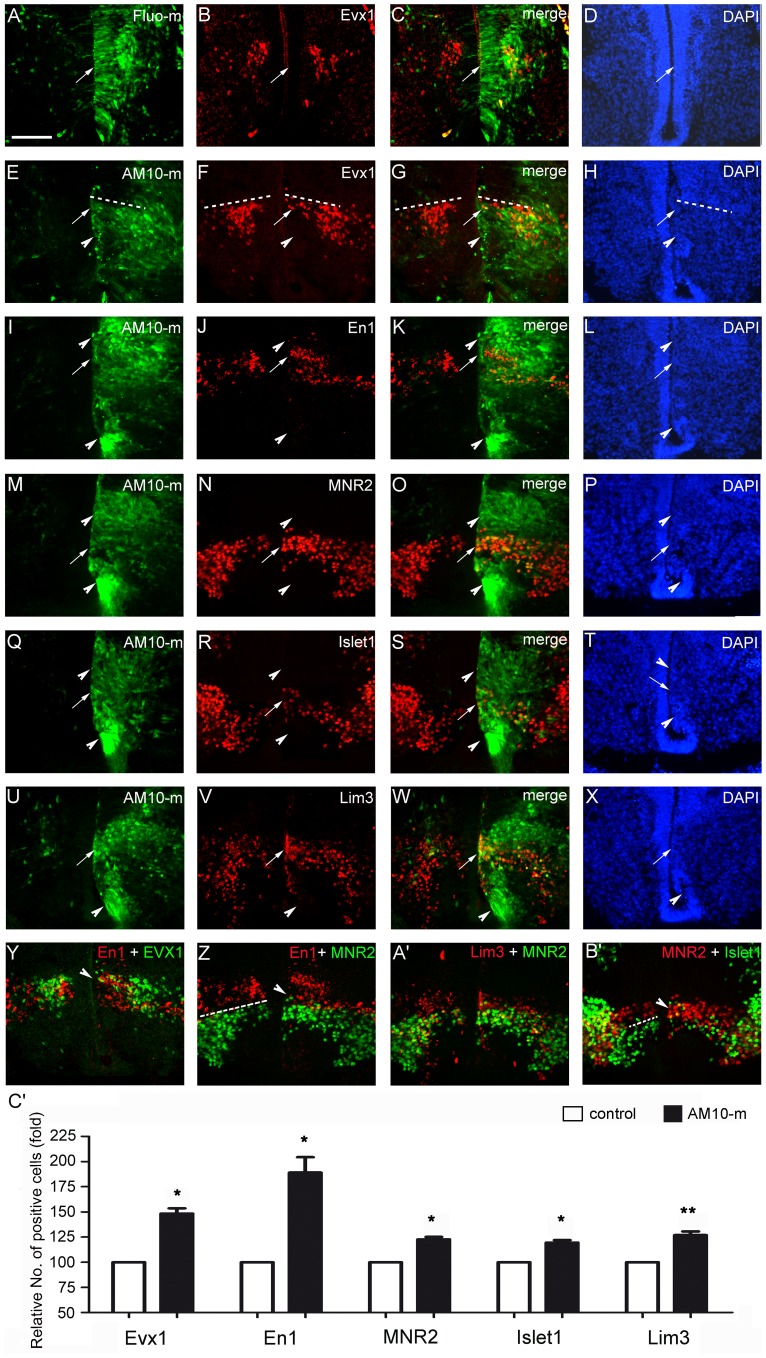
Downregulation of ADAM10 induces neuronal differentiation in the ventral spinal cord. After electroporation at E4, transverse sections at E6 were used for immunostaining. The transfected cells are marked with green fluorescence, and the control morpholinos (Fluo-m) and the untransfected side (left side) serve as controls. The immune reactive cells are stained red. Cell nuclei are labeled by DAPI (blue). (A–X) Immunohistochemistry was performed in adjacent sections with antibodies against Evx1 (A–H), En1 (I–L), MNR2 (M–P), Islet1 (Q–T), and Lim3 (U–X), respectively. Arrows indicate immune reactive cells in the prospective ventricular zone and arrowheads indicate no immune reaction in the transfected region (green). Dotted lines indicate the boundary of the alar plate and the basal plate. (Y–B’) Computer-generated overlay of neurons with different domain markers such as En1 (red) and Evx1 (green) in (Y); En1 (red) and MNR2 (green) in (Z); Lim3 (red) and MNR2 (green) in (A’); and MNR2 (red) and Islet1 (green) in (B’). Arrowheads indicate the expanded cells over the boundary into adjacent domains. Dotted lines indicate the boundary of the different domains. (C’) Quantitative data of the cell number in different domains normalized by the number of the control side, which is set to be 1. All data are presented as mean ± SEM from at least 3 independent experiments (*p<0.05, **p<0.01 compared to the control). Scale bar, 200 µm in (A) for (A–X).

In order to investigate the precise regulation of ADAM10 on cell differentiation, we evaluated the expression of postmitotic interneuron- or motoneuron-domain markers, i.e. Evx1 for V_0_ domain (Fig. 3A–H), En1 for V_1_ domain (Fig. 3I–L), MNR2 and islet1 for V_MN_ domain (Fig. 3M–P and Fig. 3Q–T), and Lim3 for V_2_ and V_MN_ domains (Fig. 3U–X), which are usually located in the mantle layer of the spinal cord. When control Fluo-mo was transfected into the spinal cord at E4, the expression patterns of the postmitotic neurons positive for different markers in the transfected (green) and untransfected (left side) side at E6 were similar, which located mainly in the mantle layer (Fig. 3A–D for Evx1; Fig. S1A–D for En1; Fig. S1E–H for MNR2; Fig. S1I–L for Islet1; and Fig. S1M–P for Lim3). However, downregulation of ADAM10 by ADAM10-mo generally promoted precocious neuronal maturation and increased the number of neurons, which were positive for the different markers. These precocious neurons even located in the prospective ventricular zone inside the transfected region from V_0_ to V_MN_ domains (arrows in Fig. 3E–X). For example, normally, MNR2-positive mature motor neurons were found at the V_MN_ domain of the mantle layer, including the motor column in the untransfected side (red, left side in Fig. 3N, O), but in the ADAM10-mo transfected regions, MNR2-positive premature motor neurons (red, Fig. 3N, O) were found even in the prospective ventricular zone (arrows in Fig. 3N, O). However, although a large ADAM10-mo transfected cells were found in the V_1_ and V_2_ domains above the V_MN_ domain (above arrowheads in Fig. 3M, O), the expression of MNR2 protein was not induced in V_1_ and V_2_ domains, suggesting that MNR2 expression was also controlled by ADAM10-independent mechanisms, e.g., Shh signaling. Quantitative results revealed that the numbers of cells expressing each individual domain marker were significantly increased in the ADAM10-mo transfected side of the V_0_ domain (Evx1, *p* = 0.015), the V_1_ domain (En1, *p* = 0.037), the V_MN_ domain (MNR2, *p* = 0.018; Islet1, *p* = 0.043), and the Lim3-positive domain (*p* = 0.03), when compared to the untransfected side (Fig. 3C’). Moreover, in order to investigate the boundary between different domains in transfected regions, color-coded overlays of the immunostaining images with the different domain markers at the same position in adjacent sections were merged by computer with the Photoshop software. The results showed that in the ADAM10-mo transfected side, the boundary between the neighboring domains disappeared and cells in different domains mixed each other when compared to the untransfected side with a clear boundary (dotted line in Fig. 3Y–B’). For example, in the transfected side (right side in Fig. 3Y–B’), the distribution of the En1-positive cells (red in Fig. 3Y) was slightly expanded up into V_0_ domain (arrowhead in Fig. 3Y) and down into V_MN_ domain (arrowhead in Fig. 3Z), while, in the untransfected control side, a sharp boundary between En1-positive and MNR2-positive cells was clearly observed (dotted line in Fig. 3Z). These data suggest that downregulation of ADAM10 promoted precocious neuronal maturation, even in the prospective ventricular zone, and caused a slight expansion of individual domain cells into the neighboring domains from V_0_ to V_MN_ domains (Fig. 3). Noted that the fluorescein-positive cells in the alar plate usually located in the mantle layer (Fig. 3E–H).

### Effects of ADAM10 Downregulation on Neural Proliferation

During spinal cord development, postmitotic neurons develop from neural progenitor cells located in the ventricular zone and migrate under the guidance of radial glial cells into the mantle layer. Since we found that the postmitotic neurons distribute even in the ventricular zone, the question is whether downregulation of ADAM10 affects neural proliferation. Therefore, we further investigated cell proliferation using the BrdU labeling method and the expression of neuronal progenitor cell markers of Pax6, NKx6.1, Pax7, and NKx2.2 by immunohistochemistry after ADAM10-mo was electroporated into the spinal cord at E4.

**Figure 4:**
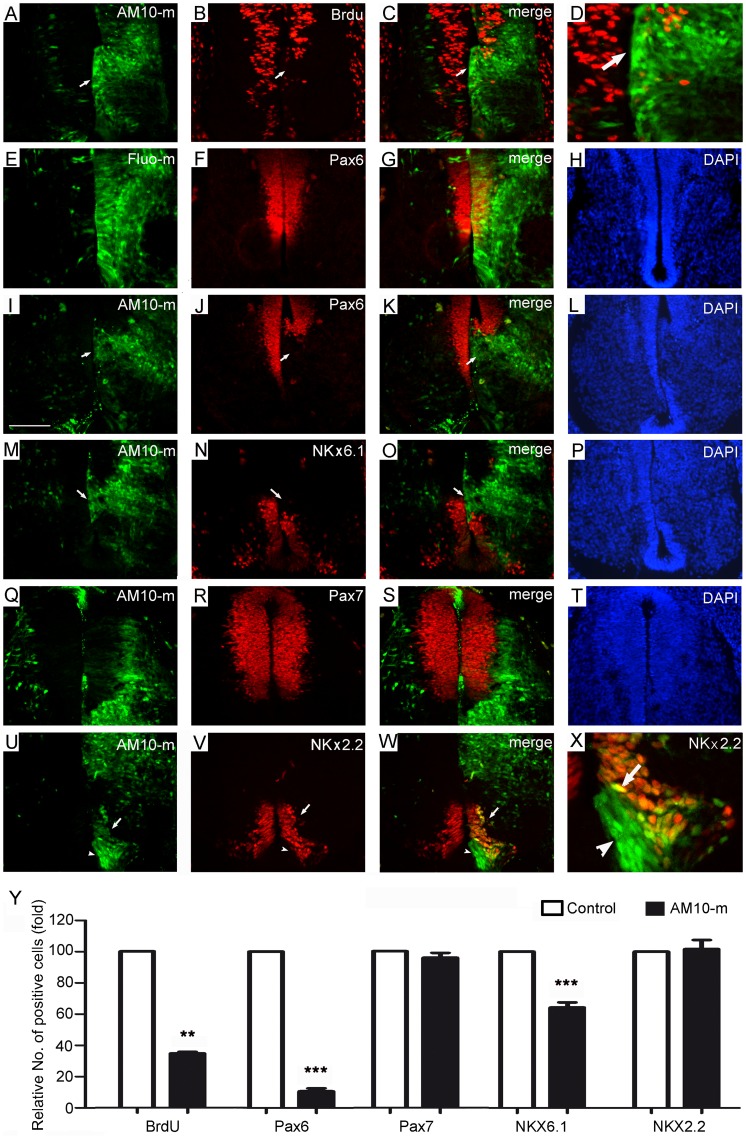
Downregulation of ADAM10 inhibits cell proliferation in the ventral spinal cord. After electroporation at E4, transverse sections were used for detection at E6. The transfected cells are marked by green fluorescence, and the control morpholinos (Fluo-m) and the untrasfected side (left side) serve as controls. The immune reactive cells are stained by red. Cell nuclei are labeled by DAPI (blue). (A–X) Immunohistochemistry was performed in adjacent sections with antibodies against BrdU (A–D), Pax6 (E–L), NKx6.1 (M–P), Pax7 (Q–T), NKx2.2 (U–X), respectively. Arrows in (A–D) indicate decrease of BrdU-labeled cells; Arrows in (I–O) indicate the immune negative region within the transfected side. (Y) Quantitative data of the cell number in different domains normalized by the number of the control side, which is set to be 1. All data are presented as mean ± SEM from at least 3 independent experiments (**p<0.01, and ***p<0.001 compared to the control). Scale bar, 200 µm in (I) for (A–W).

In ADAM10-mo transfected sections at E6, the BrdU labeling experiments revealed that the number of BrdU-positive (proliferating) cells (red in Fig. 4A–D) in the transfected region (green, arrows in Fig. 4A, C, D) was strongly decreased compared to that at the same level in the untransfected side (left side in Fig. 4A–D) and this difference was significant (*p* = 0.008).

When control Fluo-mo was transfected alone, the expression of neuronal progenitor cell markers in the transfected (right, green in Fig. 4E–H) and untrasfected (left) side was similar (Fig. 4E–H for Pax6; see Fig. S1Q–T for NKx6.1). However, when ADAM10-mo was transfected, the expression of the ventral part of Pax6 protein (arrows in Fig. 4J, K) in the basal plate, but not in the alar plate, was inhibited in the ADAM10-mo transfected side (green in Fig. 4I, K) when compared to the untransfected side (red, left side in Fig. 4J, K). Furthermore, the expression of NKx6.1 protein (arrows in Fig. 4N, O) in the basal plate, but not in the alar plate, was inhibited in the ADAM10-mo transfected side (green in Fig. 4M, O) when compared to the untransfected side (red, left side in Fig. 4N, O). Interestingly, although the fluorescein-positive cells were found inside the mantle layer of the alar plate (green in Fig. 4I, K, M, O), the expressions of Pax6 (red, Fig. 4J, K) and Pax7 (red, Fig. 4R, S) in the alar plate were not changed in the transfected side (red, right side in Fig. 4J, K and Fig. 4R, S) compared to the untransfected side (red, left side of Fig. 4K, S and Fig. 4R, S). Remarkably, although ADAM10-mo transfected cells were clearly found above the floor plate and in the V_3_ domain (green, arrowheads in Fig. 4U, W, X), the expression of NKx2.2 (arrows, Fig. 4V, W, X) in the V_3_ domain was similar between the transfected side (green in Fig. 4U, W, X) and the untransfected side (left side in Fig. 4U–W). When quantifying the number of positive cells for each individual marker, we found that the numbers of Pax6- and NKx6.1-positive cells in the transfected side were significantly decreased compared to the untransfected side (Fig. 4Y; *p* = 0.0001 for Pax6 and *p* = 0.0009 for NKx6.1), but the numbers of Pax7 and NKx2.2-positive cells in the alar plate and in the V_3_ domain was equal between both the sides, respectively (Fig. 4Y). These findings suggested that downregulation of ADAM10 specifically inhibits the certain neuronal progenitor markers in the ventral basal plate, but not in the alar plate and V_3_ domain.

### Downregulation of ADAM10 Drives Differentiation of Radial Glial Cell

**Figure 5:**
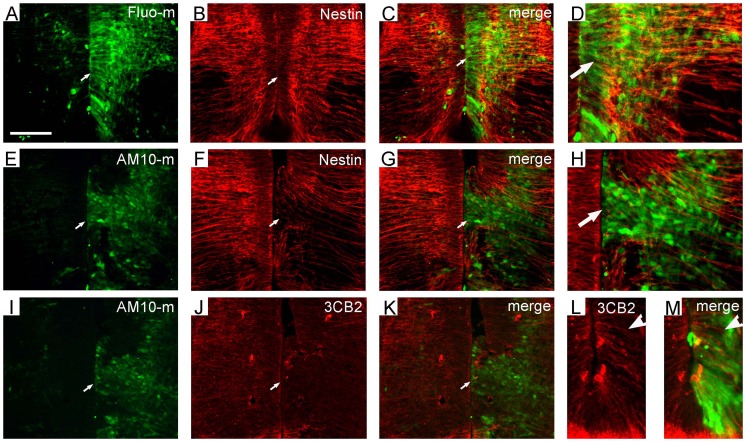
Downregulation of ADAM10 drives differentiation of radial glial cells in the ventral spinal cord. After electroporation at E4, transverse sections were used for detection at E6. The transfected cells are marked by green fluorescence and the control morpholinos (Fluo-m) and the untransfected side (left side) serve as controls. The immune reactive cells are stained by red. Cell nuclei are labeled by DAPI (blue). Immunohistochemistry was performed in adjacent sections with antibodies against Nestin (A–H) and vimentin (3CB2; I–M), respectively. Arrows in A–D indicate transfected regions (green) and in E–K the inhibition of nestin (E–H) and vimentin (I–K) in the transfected region. Noted that no change of viementin expression in the transfected region above the floor plate is found (arrows in L, M). Scale bar, 200 µm in (A) for (A–K).

To further investigate the possible reasons for morphological alteration in ADAM10-mo electroporated regions, we evaluated the expression of radial glial cell markers, e.g., nestin and vimentin, in the electroporated embryos. When control Fluo-mo was transfected alone into the spinal cord at E4, the expression of nestin protein (red in Fig. 5B–D) in the transfected side (green in Fig. 5A–D) and untransfected sides (left in Fig. 5A–D), as well as vimentin protein (see Fig. S1U–X), was similar. However, when ADAM10-mo was transfected, the expressions of nestin (red in Fig. 5F–H) and vimentin (red in Fig. 5J, K) in the transfected region (arrows, green in Fig. 5E–K) of the basal plate were decreased, but not in the V_3_ domain (e.g., Fig. 5L, M for vimentin).

Taken together, our data suggested that downregulation of ADAM10 specifically drive differentiation of radial glial cells located in the ventricular zone of the specific domains of the basal plate into neurons.

### Metalloprotease Domain in ADAM10 is Essential for ADAM10 Function

ADAM10 is a type I transmembrane protein and possesses a metalloprotease domain and a disintegrin domain with multiple functions. To examine whether proteolytic shedding is necessary for the ADAM10 function during the spinal cord development, dn-ADAM10-me plasmid was used [12]. After transfected into human embryonic kidney 293 (HEK) cells, the protein of the dn-ADAM10-me was highly expressed in vitro, as revealed by Western blot analysis, and was sizably comparable to the endogenous and ADAM10 overexpression with pCAGGS-ADAM10 plasmid (data not shown), suggesting that the construct of dn-ADAM10-me was correctly expressed and suitable for applying for overexpression in vivo by electroporation.

**Figure 6:**
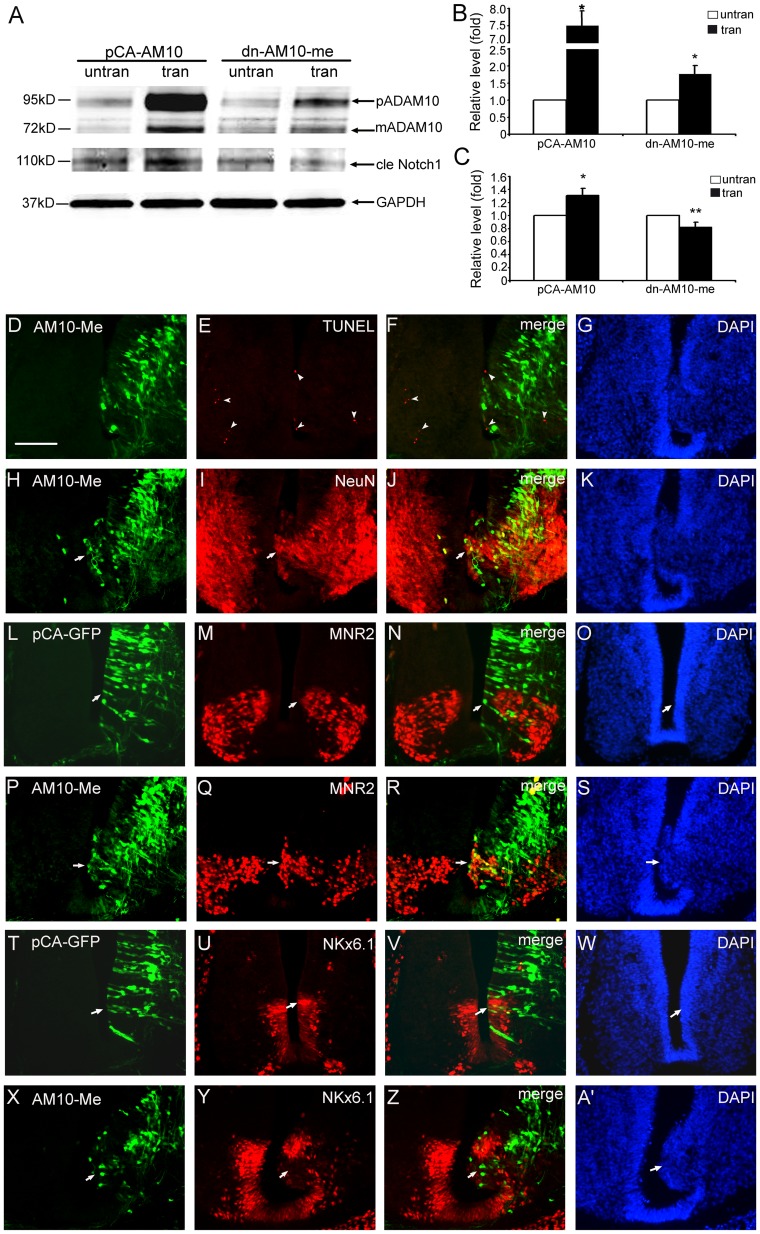
Overexpression of a dominant-negative ADAM10 mutated in the metalloprotease domain (dn-ADAM10-me) promotes neuronal differentiation in the developing spinal cord. After electroporation at E4, transverse sections at E6 were used for immunostaining. The dn-ADAM10-me (AM10-me) transfected cells are marked by green fluorescence and pCAGGS-GFP (pCA-GFP) transfection and untransfected side (left side) serve as controls. The immune reactive cells are stained by red color. Cell nuclei are labeled with DAPI (blue). (A–C) Representative Western blots (A) and semi-quantitative Western blot analyses (B, C) of ADAM10 protein, including a pre-mature (pADAM10) and a mature (mADAM10) form, and the cleaved Notch1 (cle Notch1) in the transfected (tran) and untransfected side (untran) of pCAGGS-ADAM10 (pCA-AM10) or dn-ADAM10-me (AM10-me) transfected embryos. GAPDH is used as a loading control. The amount of ADAM10 and cle Notch1 protein is normalized by the number of the control side, which is set to be 1. All data are presented as mean ± SEM from at least 3 independent samples (*p<0.05, **p<0.01 compared to control). (D–G) Apoptotic cells (red, arrowheads) measured by TUNEL assay. (H–A’) Immunostaining using antibodies against NeuN (H–K), MNR2 (L–S), and NKx6.1 (T–A’), respectively. Arrows in (H–K) and (P–S) indicate ectopic immune reaction in the prospective ventricular zone of the transfected region (green); in (L–O) and (T–W) no change in the transfected region (green); in (X–A’) decrease of endogenous NKx6.1 expression. Scale bar, 200 µm in (D) for (D–A’).

When dn-ADAM10-me or pCAGGS-ADAM10 plasmid was transfected into the spinal cord of chicken embryos, and two days after electroporation at E6, the separated transfected or untransfected spinal cords were collected for Western blot analysis. The pCAGGS-ADAM10 for ADAM10 overexpression was used as a positive control. The quantitative Western blot results showed that ADAM10 protein, including the pre-mature (pADAM10 in Fig. 6A) and mature (mADAM10 in Fig. 6A) form, was significant increased in the transfected side (tran, Fig. 6A, B) compared to the untransfected side (untran, Fig. 6A, B) in the pCAGGS-ADAM10 or dn-ADAM10-me transfected embryos (Fig. 6A, B; *p* = 0.014 and 0.022, respectively). ADAM10 has been reported to be responsible for shedding of Notch receptors by its proteolytic function [39]. To detect whether the proteolytic function in the mutant ADAM10 protein was defect, the cleaved Notch1– a Notch1 intracellular domain (NICD), was investigated by Western blots using Notch1 antibody. The quantitative data showed that the amount of the cleaved Notch1 protein was significantly increased in the transfected side of pCAGGS-ADAM10 transfected embryos (pCA-AM10, tran; Fig. 6A, C; *p = *0.033), but decreased of the dn-ADAM10-me transfected embryos (dn-AM10-me, tran; Fig. 6A, C; *p* = 0.0096), when compared to the control untransfected side (untran, Fig. 6A, C), suggesting that the proteolytic function in the mutant ADAM10 was defect.

Furthermore, immunohistochemistry was performed to investigate the changes of different markers under dn-ADAM10-me transfection. When the control pCAGGS-GFP plasmid was transfected alone into the spinal cord at E4, the protein expressions of NeuN (data not shown), MNR2 (red, Fig. 6L–O), and NKx6.1 (red, Fig. 6T–W) at E6 between the transfected side (green in Fig. 6L–O, T–W) and the untransfected side (left side in Fig. 6L–O, T–W) was similar. When dn-ADAM10-me was transfected, TUNEL assay showed that the number of apoptotic cells (red, arrowheads in Fig. 6E, F) in the transfected side (green, Fig. 6D, F) was not different from that in the untransfected side (left in Fig. 6D–G). However, in the transfected region (GFP-positive; green, Fig. 6H, P, X), the expressions of NeuN (red, Fig. 6I, J), MNR2 (red, Fig. 6Q, R), NKx6.1 (red, Fig. 6Y, Z), and other interneuron markers, e.g., Islet-1 (data not shown), were induced or inhibited in the prospective ventricular zone (arrows in Fig. 6H–K, P–S, X–A’), and the numbers of the MNR2- and NKx6.1-positive cells were increased or decrease, respectively, when compared to the untransfected side (left in Fig. 6O–R, Y–Z; data not shown). These results mimicked the phenomena of ADAM10-mo transfection (Figs. 2–4) and indicated that the metalloprotease domain was essential for the normal function of ADAM10 in regulating the development of the spinal cord.

### Downregulation of ADAM10 Perturbs Notch Signaling Pathway

**Figure 7:**
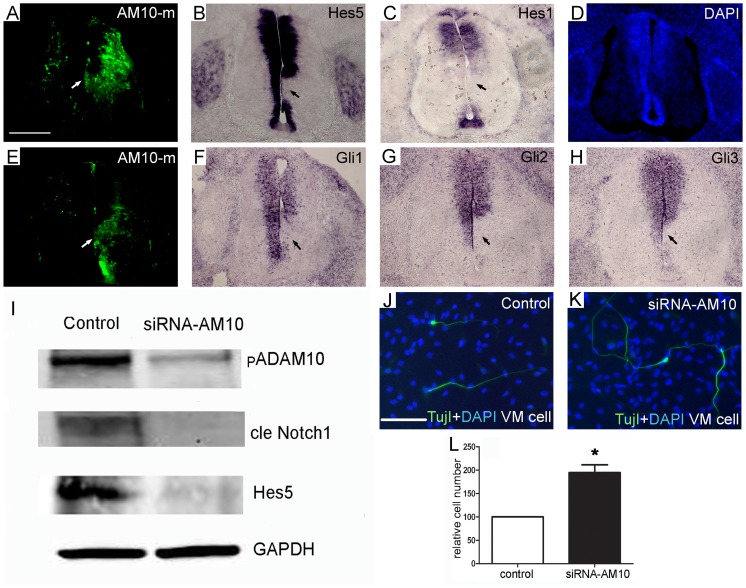
Inhibition of ADAM10 affects Notch targeted Hes5 and Gli genes in the ventral spinal cord (A–H) and in vitro cultured cell system (I–M). (A–H) After electroporation at E4, transverse section at E6 was used for in situ hybridization. The ADAM10-mo transfected cells are green and the left side serves as a control. In situ hybridization using the antisense probes for Hes5 (B), Hes1 (C), Gli1 (F), Gli2 (G), and Gli3 (H) are labeled purple and cell nuclei are blue. Arrows indicate the downregulation of mRNA expression (purple color) in the transfected region (green). (I) Western blot analysis reveals that premature ADAM10 (pADAM10), the cleaved Notch1 (NICD), and Hes5 proteins are decreased in the ADAM10 siRNA transfected human neural progenitor cells at differentiation day 3, when compared to the control group (n = 2). GAPDH was used as a loading control. (J–L) Three days after differentiation, cells were stained against βIII-tubulin using antibody Tuj1 (green) in control siRNA (J) and ADAM10 siRNA transfected cells (K). Cell nuclei are labeled with DAPI (blue). Quantitative analyses reveal that the number of Tuj1-positive cells (L) significantly increase in the ADAM10 siRNA transfected cells compared to the control group (*p<0.05). Abbreviations: cle Notch1, cleaved Notch1. Scale bars, 200 µm in (A) for (A–H); 100 µm in (J) for (J, K).

The Notch signaling pathway plays a key role in the neural cell fate determination during embryonic development [9]. Previous studies have shown that Notch receptors and their related genes are expressed in the neuroepithelium of the developing chicken spinal cord [40], [41]. Interestingly, ADAM10 is also expressed strongly in the neuroepithelium of the spinal cord at early stages of the chicken embryo [14] and fulfils important function in shedding of Notch receptors and their ligands [39], which are involved in cell fate decision. This relationship between ADAM10 and Notch signaling led us to test whether ADAM10 regulates Notch signaling in the developing spinal cord. To address this question, expression patterns of the Notch target genes – Hes1 and Hes5 [42], [43] were investigated after ADAM10 was downregulated. When ADAM10-mo was transfected into the spinal cord (green, Fig. 7A), in situ hybridization analyses showed that the mRNA expression of Hes5 (arrow in Fig. 7B), but not Hes1 (arrow in Fig. 7C), was partially inhibited in the basal plate of the ADAM10-mo transfected region. Furthermore, the downregulation of ADAM10 on the Notch receptor was investigated by in vitro cell cultured system at 3 days after differentiation of human neural progenitor ReNcell VM cells. Western bolt analyses showed that the amount of ADAM10 protein was decreased in pGSHIN2-ADAM10 siRNA transfected cells, when compared to the control pGSHIN2-GFP group (Fig. 7I). The protein amount of NICD and its targeted protein Hes5 in the whole cell lysate was also decreased in the siRNA transfected cells (Fig. 7I). Furthermore, immunocytochemistry data showed that ADAM10 downregulation increased the number of Tuj1-positive cells in pGSHIN2-ADAM10 siRNA transfected cells, when compared to the control siRNA vector (Fig. 7J–L).

Additionally, gradient Shh concentration from the ventral to dorsal plays an important role in the dorso-ventral patterning of the spinal cord [3]. Therefore, we also evaluated the target genes of Shh, the transcription factors Gli1, Gli2 and Gli3, in the ADAM10-mo transfected regions. The results showed that the expression patterns of Gli1, Gli2 and Gli3 were partially inhibited in the ADAM10-transfected regions (arrows in Fig. 7E–H), suggesting that ADAM10 downregulation also perturbed the specific Gli genes, which are responsible for the dorso-ventral patterning in the developing spinal cord [3].

## Discussion

In this study, we provide crucial evidence to support the notion that ADAM10 plays an important role in the development of the chicken spinal cord. We found that downregulation of ADAM10 protein promotes differentiation of neural progenitor cells and radial glial cells into neurons in the certain domains of the basal plate, resulting in the premature development of neurons, even in the prospective ventricular zone, and increases the number of neurons in the ADAM10-downregulated regions (Figs. 2–5). We also found through dominant negative experiments that the effect of ADAM10 on the neuronal differentiation is mainly mediated by its metalloprotease domain, which is involved in the proteolytic processing of Notch receptors (Fig. 6) [38], [44], [45]. Furthermore, in vitro cell culture experiments showed that downregulation of ADAM10 by siRNA interference increases the number of the Tuj1-positive cells (Fig. 7), confirming that ADAM10 downregulation improves neuronal differentiation.

Previous studies have indicated that ADAM10 controls neurogenesis and is essential for the development of the central nervous system (CNS) [17], [23], [46]. For example, ADAM10 is expressed strongly in the neuroepithelial layer of the embryonic CNS [13], [14], [16], [47]. Inhibition of the ADAM10 protein in *Xenopus* by dominant-negative ADAM10 lacking protease activity leads to overproduction of primary neurons [38]. Furthermore, conditional deletion of ADAM10 in the neural progenitor cells results in promoting neuronal differentiation in the brain and disturbs the normal cerebral cortex [23]. Taken together, these data suggest a role of ADAM10 in the regulation of neurogenesis and neuronal differentiation.

The Notch signaling pathway plays an important role in cell fate decision and regulates the maintenance of neural progenitor subtypes, especially of the ventral spinal cord, during development of the spinal cord [11], [48], [49]. Overexpression of Notch receptor in *Xenopus* leads to a significant increase of precursor cells in the neural tube and inhibits cell differentiation [50]. By contrast, disruption of Notch signaling causes accelerated neuronal differentiation in the ventral spinal cord accompanied by a reduced expression of Hes5 [48], [51]. ADAM10, as a Notch sheddase at the S2 site, is essential for activation of the Notch signaling pathway [38], [44], [45]. ADAM10-deficient mice exhibit a downregulation of Notch1 protein and its target gene Hes5 in the brain [11], [23]. The conditional Notch1-mutant mice show interesting phonomena: predominant defects in the specifically basal plate of the spinal cord, including a disappearance of the ventricular zone and an increase of neuronal number in the ventral spinal cord [11]. Consistent with these findings, we found that downregulation of ADAM10 in the developing spinal cord inhibits the expression of Hes5 in the ventral spinal cord (Fig. 7B), but not in the floor plate and dorsal region of the spinal cord. ADAM10 has no effect on Hes1 expression, which is not endogenously expressed in the ventral part at the embryonic stage investigated (Fig. 7C). Furthermore, dominant negative ADAM10 mutated in the metalloprotease domain induces the same phenomena as the ADAM10-mo (Fig. 6), suggesting that ADAM10 regulates the maintenance of progenitor cells in the developing spinal cord, possibly via a proteolytic effect on Notch signaling and affecting Hes5.

In this study, we found that the distribution patterns of the fluorescence-positive (transfected) cells in the control groups with Fluo-mo and pCAGGS-GFP transfection are different with that in the experimental groups with ADAM10-mo and dn-ADAM10-me transfection. In control groups, the fluorescence-positive cells distribute in the ventricular zone and mantle layer of both the basal and alar plate, showing a normal differentiation and migration process. However, for the experimental groups, in the basal plate, ADAM10 downregulation in the fluorescence-positive (transfected) cells drives precocious differentiation of these neural progenitor cells. The ‘earlier’ fluorescence-positive differentiated neurons can migrate under the guidance from radial glial cells into the mantle layer, but the ‘later’ fluorescence-positive differentiated neurons may stay in the ventricular zone without any guidance from the radial glial cells or even these neurons directly differentiated from the radial glial cells. Therefore, the fluorescence-positive neurons distribute in both the mantle layer and ventricular zone of the basal plate in the experimental groups (Figs. 1–6); in the alar plate, Notch signalling may take less effect on cell development as shown by a Notch1 knock-out mouse model [11], or ADAM10 function in radial glial cells may be redundant, because ADAM17, which is strongly expressed in the ventricular zone of chicken spinal cord from E3 onwards and predominant at E6 [14], can also activate Notch signaling by shedding the same S2 site in a context dependent manner [52]–[54]. In C. elegans, cell fate determination mediated by LIN-12/Notch is controlled by SUP-17 and ADM-4, orthologs of mammalian ADAM10 and ADAM17, which are functionally redundant [55]. Therefore, Notch signaling in the radial glial cells of the alar plate can be activated by ADAM17 shedding, resulting in the maintenance of the radial glial cells in the ventricular zone (Fig. 5). On the other hand, ADAM10 downregulation in the neural progenitor cells induces premature formation of neurons, which migrate into the mantle layer under the guidance of the radial glial cells. Therefore, we found seldom fluorescence-positive neurons locating in the ventricular zone of the alar plate in the experimental groups (Figs. 1–6).

Interestingly, in the ADAM10 downregulation region the expression of Gli1, Gli2 and Gli3 in the ventricular zone of the specific ventral domains are also inhibited. One possible explanation for this phenomenon is that downregulation of ADAM10 drives neural progenitor cells and radial glial cells differentiation, as indicated by decreases of nestin- and viementin-expression cells (Fig. 5) and by an increase of NeuN-positive cells (Fig. 2) in the ventricular zone. Since Gli genes are mainly expressed in the neural progenitor cells of the ventricular zone, downregulation of ADAM10 induces premature of neurons in the prospective ventricular zone, resulting in the disappearance of the neural progenitor cells and, under this condition, the expression of Gli genes disappears. Whether ADAM10 directly regulates the expression of Gli genes expressing in the ventricular zone during spinal cord development should be further investigated.

## Supporting Information

Figure S1 **Control morpholinos have no effect on** **the development of the spinal cord.** After control morpholinos (Fulo-m) weren electroporated into chicken embryos at E4, transverse sections were used for detection of different markers at E6. The Fluo-m transfected (positive) cells are labeled as green in the transfected side (right side) and the untransfected sides in the left serve as controls. The immune reactive cells are stained by red. Cell nuclei are labeled by DAPI (blue). (A–X) Immunohistochemistry was performed in adjacent sections with antibodies against En1 (A–D), MNR2 (E–H), Islet1(I–L), Lim3 (M–P), NKx6.1 (Q–T), and 3CB2 (U–X), respectively. Scale bar, 200 µm in (A) for (B–P) and in (Q) for (R–T), 50 µm in (U) for (V–X).
(TIF)

Figure S2 **Separation of the electroporated spinal cord.** A white light image is merged with a fluorescent image to show the separated electroporated spinal cord from dorsal view. Two days after electroporation with different morpholinos or plasmids (e.g., here pCAGGS-ADAM10 together with pCAGGS-GFP is transfected), the electroporated fresh spinal cord was separated into transfected (tran, green) and untransfected side (untran) under the fluorescent microscope for further measurement by Western blots.
(TIF)
