# Supplementary figures and images for: ADAM10 Negatively Regulates Neuronal Differentiation during Spinal Cord Development
Source: PLoS One. 2014 Jan 3;9(1):e84617. doi: 10.1371/journal.pone.0084617 (PMC3880303; doi:10.1371/journal.pone.0084617)

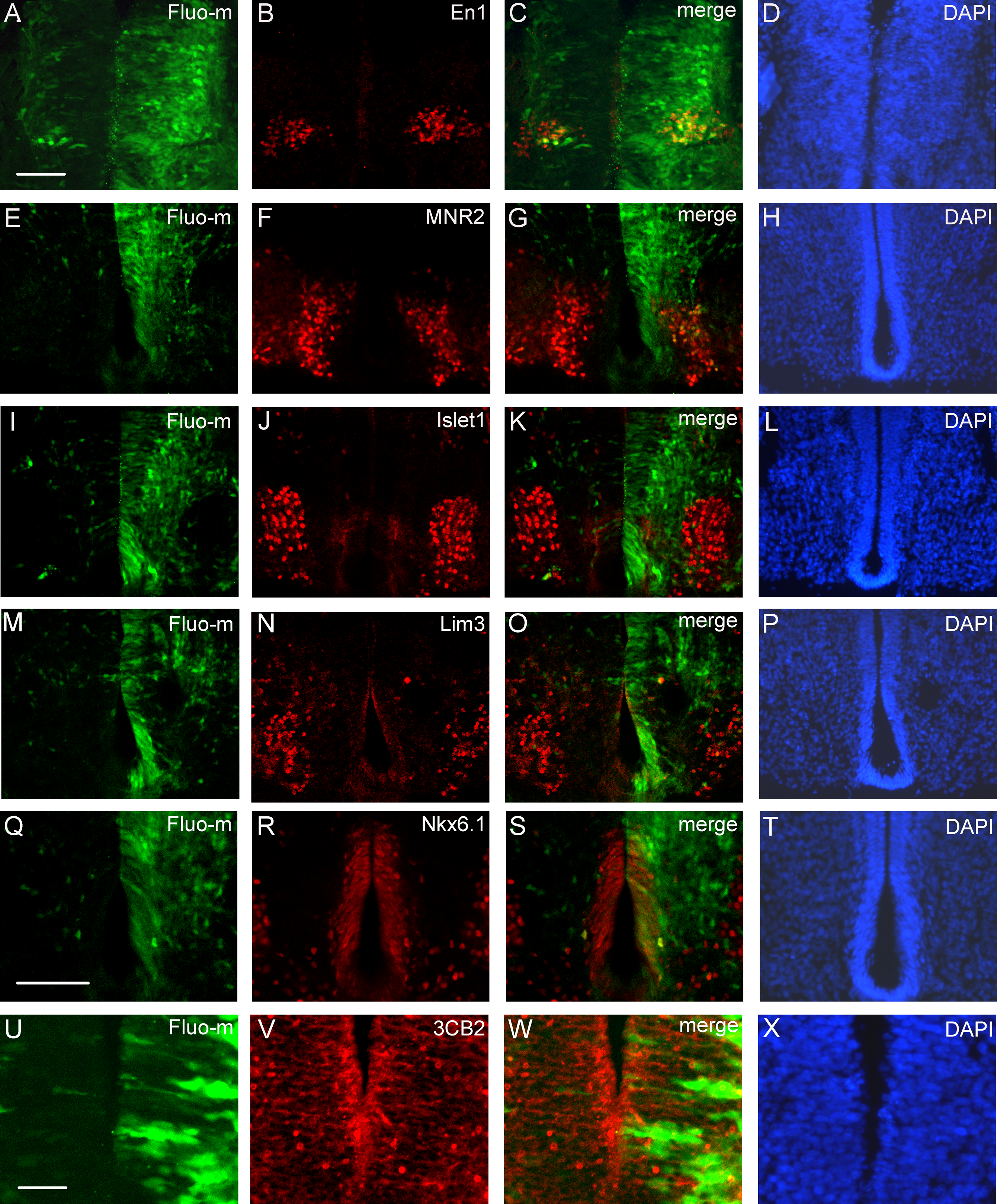

Supplement: Figure S1 — Control morpholinos have no effect on the development of the spinal cord. After control morpholinos (Fulo-m) weren electroporated into chicken embryos at E4, transverse sections were used for detection of different markers at E6. The Fluo-m transfected (positive) cells are labeled as green in the transfected side (right side) and the untransfected sides in the left serve as controls. The immune reactive cells are stained by red. Cell nuclei are labeled by DAPI (blue). (A–X) Immunohistochemistry was performed in adjacent sections with antibodies against En1 (A–D), MNR2 (E–H), Islet1(I–L), Lim3 (M–P), NKx6.1 (Q–T), and 3CB2 (U–X), respectively. Scale bar, 200 µm in (A) for (B–P) and in (Q) for (R–T), 50 µm in (U) for (V–X). (TIF) [file pone.0084617.s001.tif]

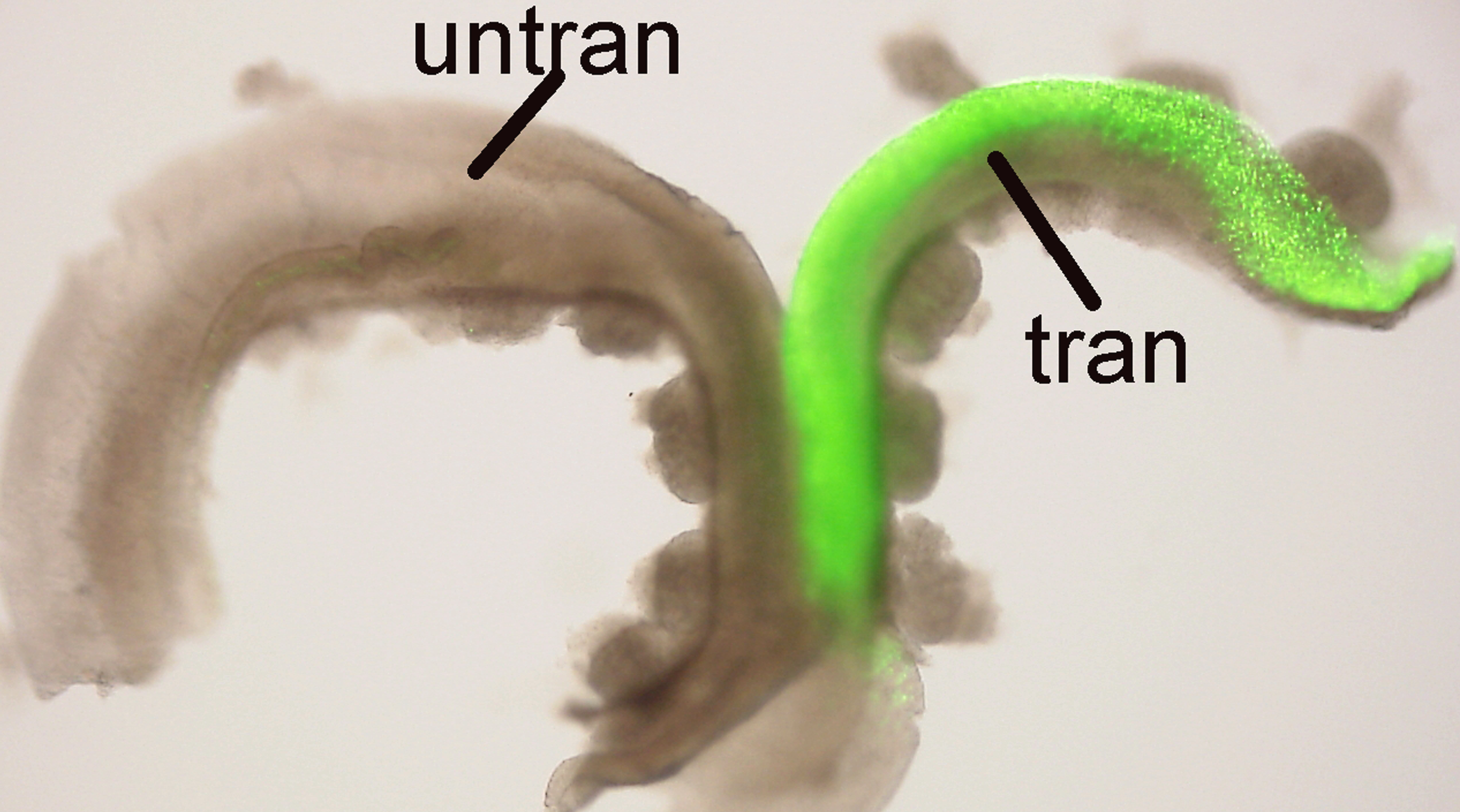

Supplement: Figure S2 — Separation of the electroporated spinal cord. A white light image is merged with a fluorescent image to show the separated electroporated spinal cord from dorsal view. Two days after electroporation with different morpholinos or plasmids (e.g., here pCAGGS-ADAM10 together with pCAGGS-GFP is transfected), the electroporated fresh spinal cord was separated into transfected (tran, green) and untransfected side (untran) under the fluorescent microscope for further measurement by Western blots. (TIF) [file pone.0084617.s002.tif]
